# SpChipADF: An Architecture Design Framework for Radar Signal Processing Hardware Accelerators

**DOI:** 10.3390/mi17050535

**Published:** 2026-04-27

**Authors:** Huan Wang, Shu Yang, Zhen Chen, Haoyu Sun, Yang Shen, Hang Li, Zhiyu Jiang, Yanlei Li, Xingdong Liang

**Affiliations:** 1 National Key Laboratory of Microwave Imaging Technology, Aerospace Information Research Institute, Chinese Academy of Sciences, Beijing 100190, China; wanghuan21a@mails.ucas.ac.cn (H.W.); yangshu22@mails.ucas.ac.cn (S.Y.); chenzhen18@mails.ucas.ac.cn (Z.C.); sunhaoyu24@mails.ucas.ac.cn (H.S.); shenyang@aircas.ac.cn (Y.S.); lihang212@mails.ucas.ac.cn (H.L.); jiangzhiyu22@mails.ucas.ac.cn (Z.J.); 2 School of Electronic, Electrical and Communication Engineering, University of Chinese Academy of Sciences, Beijing 100094, China

**Keywords:** UAVs, SWaP, edge-side signal processing, algorithm-hardware co-design, reconfigurable architecture, SAR imaging

## Abstract

Lightweight Unmanned Aerial Vehicles (UAVs) have limited space, low payload capacity, and constrained power supply capabilities. Therefore, their payloads are constrained by size, weight, and power (SWaP). Thus, designing edge-side signal processing architectures for the payloads of UAVs faces severe challenges. Traditional ASIC design based on manual optimization struggles to meet the demands of low latency and low resource occupancy in edge-side applications. To address this challenge, this paper proposes a signal processing hardware accelerator architecture design framework with algorithm-hardware co-design. The framework employs a cross-level dataflow graph representation to formally capture task characteristics. Reconfigurable dataflow templates and reusable operator IP components are systematically constructed based on this representation. Through multi-objective design space exploration, the framework achieves Pareto-optimal mapping from algorithmic specifications to hardware implementations. Finally, automatic generation of top-level hardware descriptions enables rapid FPGA-based prototyping and functional validation. Taking synthetic aperture radar (SAR) imaging as a study example, compared with non-reconfigurable architectures, this scheme reduces the equivalent gate count by 51.4% without increasing processing latency. Compared with a conventional reconfigurable dataflow architecture, the design improves energy efficiency from 12.8 MS/J to 16.0 MS/J, representing a 25.4% enhancement, while also scaling the supported data processing size by a factor of 4×. It provides a high-performance and scalable hardware acceleration solution for lightweight edge-side computing platforms.

## 1. Introduction

**Figure 1:**
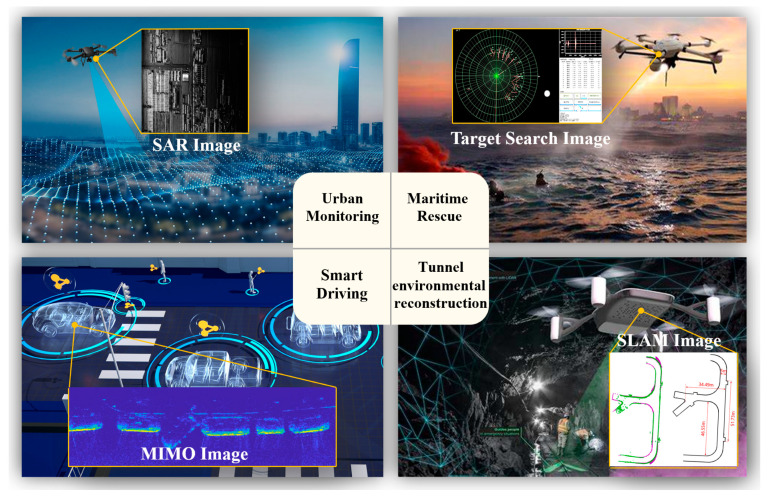
Edge-Side Radar Signal Processing Application Scenarios.

Due to their all-weather and all-day characteristics, real-time radar systems play a crucial role in various applications such as urban monitoring, maritime rescue, intelligent driving, and mine tunnel environmental reconstruction [1,2], as shown in Figure 1. Among the subsystems of a radar, the signal processing module is key, as it processes massive amounts of echo data to extract actionable information. In recent years, advances in microelectronics have enabled continuous miniaturization of real-time radar systems, and their deployment platforms have shifted from large aircraft and ground-based stations to lightweight vehicles such as UAVs and automobiles. In these emerging scenarios, the demand for high-throughput and complex signal processing computations increasingly conflicts with the SWaP constraints of lightweight platforms.

For real-time signal processing on lightweight platforms, the mainstream processors include central processing units (CPU), digital signal processors (DSP), graphics processing units (GPU), field-programmable gate arrays (FPGA), and application-specific integrated circuits (ASIC) [3].

The CPU adopts the von Neumann architecture, which is suitable for general-purpose computing. However, due to the large overhead of redundant instructions and low parallelism, it is difficult to meet the performance and power consumption requirements. The DSP adopts the Harvard architecture, which increases the bus width to separate instructions and data, reducing bus competition. In on-chip cache design, the cache is divided into instruction cache and data cache, supporting parallel pre-fetching of instructions and data. The DSP integrates efficient floating-point computing units and high-speed caches and improves the processing speed through multi-core parallel processing, making it suitable for compute-intensive applications. Wang et al. from TI implemented RDA on the 8-core DSP TMS320C6678, with a peak power consumption of 10 W, and completed SAR imaging processing of 4 K × 4 K size in 0.25 s [4]. However, its serial processing characteristic leads to performance improvement relying on increasing the main frequency and the number of cores, which limits further improvement of energy efficiency.

GPU was initially used for graphics rendering and numerical analysis. Later, its powerful parallel computing capabilities led to widespread adoption in artificial intelligence computing and radar signal processing. A GPU consists of multiple Streaming Multiprocessors (SMs), and each SM contains multiple Streaming Processors (SPs). Thus, a GPU can be viewed as an enhanced version of a DSP. For edge-side SAR scenarios, embedded GPUs are more suitable due to their high energy efficiency. In 2022, Yang et al. implemented the Chirp Scaling Algorithm (CSA) on the Jetson Nano embedded GPU platform and completed the processing of 8 K × 8 K data in 5.86 s [5]. In 2024, Yang et al. realized video SAR imaging processing based on the Range-Doppler Algorithm (RDA) on the Jetson AGX Orin embedded GPU platform. They could complete the SAR imaging processing of 2 K × 2 K size in 0.215 s, with a frame rate of 5 Hz [6]. GPUs enhance the parallel processing scale by increasing the number of processing cores. However, each processing core still uses an instruction-pipelined serial processing method, which results in relatively high redundant power consumption.

FPGA has long held a prominent position in the field of signal processing, owing to its reconfigurability and hardware programmability. In 2017, M. Wielage et al. compared the energy efficiency and computing speed of the Virtex Ultrascale+ FPGA and the Jetson TX2 GPU based on the Back-Projection (BP) algorithm [7]. The results showed that the Virtex Ultrascale+ FPGA had 6.5 times higher energy efficiency and 4.3 times higher speed than the TX2 GPU. With the improvement in FPGA’s performance, its advantages in SAR imaging processing have become increasingly apparent. In 2024, Lin et al. utilized High Bandwidth Memory (HBM) and a 14-nm FPGA to implement a pipelined spaceborne SAR imaging computing architecture [8]. Based on CSA, they completed the focusing processing of 8 K × 32 K SAR data in 1.35 s. FPGA has hardware-programmable characteristics, enabling flexible reconfiguration for different functions and hardware optimization. FPGAs are suitable for parallel pipelined processing. However, their clock frequency is not high, and they are limited by hardware resources, so the level of energy efficiency improvement is limited.

Compared with FPGA, ASIC can significantly improve the processing performance and energy efficiency of specific functions. In 2015, Broich et al. proposed a soft-core dataflow computing architecture for radar signal processing (RSP), which improves the utilization of computing resources through instruction-level parallelism and software pipelining [9]. In 2016, Feng et al. put forward a reconfigurable RSP architecture based on floating-point operations, which improves computational parallelism and resource utilization by designing a reconfigurable processing array [10]. In 2022, Wang et al. designed a reconfigurable Processing Element (PE) unit array with high-width Single Instruction Multiple Data (SIMD), and proposed a reconfigurable dataflow architecture [11]. This architecture improves energy efficiency by 5.3 times compared with the NVIDIA Tesla V100 GPU, and completes the focusing processing of 8 K × 8 K SAR data within 3 s. However, ASIC has high costs and lacks flexibility, so it cannot meet the different functional requirements of various application scenarios.

**Figure 2:**
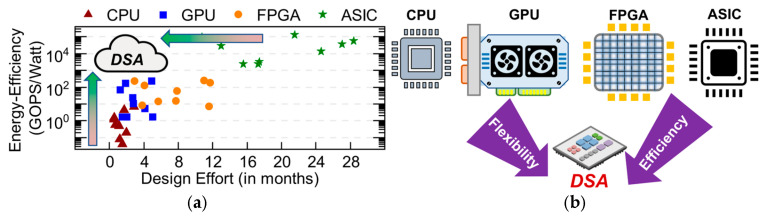
Analysis of CPU, GPU, FPGA, and ASIC c0haracteristics. (**a**) Trend chart of energy efficiency and design effort for applications implemented on CPU, GPU, FPGA, and ASIC. (**b**) Diagram illustrating the combination of flexibility and processing efficiency of DSA with CPU, GPU, FPGA, and ASIC.

Figure 2a illustrates the development trends of energy efficiency and design effort for CPU, GPU, FPGA, and ASIC [12]. It can be seen that for specific application tasks, ASIC has the highest energy efficiency but the longest design cycle, while the CPU has the lowest design effort but the lowest processing efficiency. Single-type processors can no longer meet the requirements of multi-function real-time processing for specific domain tasks on lightweight platforms. In recent years, Domain Specific Architecture (DSA) has developed rapidly. As an instance of heterogeneous architecture, DSA efficiently integrates general-purpose processors with dedicated hardware accelerators, improving energy efficiency while maintaining programming flexibility. From Figure 2b, it can be seen that compared with other types of processors, DSA can combine flexibility and processing efficiency. For various functions in the signal processing specific domain, we can in future work extract the bottlenecks that limit the improvement of processing energy efficiency and design corresponding DSAs to form high-energy-efficiency heterogeneous computing architectures.

DSA can be implemented through high-density integration technologies such as System on Chip (SoC) and System in a Package (SiP). However, SoC has high requirements for process technology, limited area, high cost, and low yield. Compared with SoC, SiP can realize large-scale integration of multiple heterogeneous chiplets by using integrated packaging technology. It can integrate different chiplets within a shorter design cycle and is an important technical route for the continuation of Moore’s Law in the future. Chiplet design is divided into two stages: architecture design and back-end implementation. The architecture design stage is mainly responsible for determining the functions of the chiplet and the top-level operational circuit structure. The back-end stage of chip design is responsible for completing the layout and routing of multiple chiplets and multi-physical field simulation. Traditional EDA toolchains are still applicable to this process, and there are also dedicated tools for 3D integrated circuits, such as Cadencia Integrity [13]. Although physical integration technologies have been relatively mature [14,15,16], the corresponding architecture design tools remain underdeveloped.

For DSA design oriented to signal processing, relevant research work has been carried out in the industry. Reference [17] proposed an integrated design method that combines the Synchronous Data Flow (SDF) model with Application-Specific Instruction-set Processor (ASIP) customization, aiming to optimize embedded systems for signal processing applications. However, since this scheme is mainly based on ASIP architecture design, it is difficult to maximize energy efficiency for specific domain applications. References [18,19] developed automated techniques to identify cross-application common code sequences and map them to custom instructions of reconfigurable Special Function Units (SFU). They proposed a heuristic method to reduce the search space of domain-specific instructions and prioritize the optimal solution across applications. Nevertheless, this method can only accelerate existing applications and struggles to optimize the data flow of the applications themselves. References [20,21] automatically generated the RTL descriptions of chiplets through high-level synthesis, which can significantly improve the design efficiency of DSA based on chiplets. However, they lack optimization of the top-level computational structure and find it difficult to achieve the optimal energy efficiency at the top-level architecture. References [22,23] conducted structured modeling of dataflow tasks and mapped the dataflow tasks to the processors in the heterogeneous architecture in an optimal way. Yet, they find it hard to directly map these to circuit architectures.

Signal processing systems on lightweight platforms face dual challenges of achieving low-latency and low-resource computing [24,25,26], while current hardware accelerator architectures lack flexibility and design methodologies remain under-developed. At the top-level architecture, DSAs based on advanced packaging technologies represent a feasible solution that balances flexibility and processing efficiency. However, for the design of hardware accelerators within DSAs, while High-Level Synthesis (HLS) can translate high-level design languages into RTL, lowering the programming barrier, its hardware constraints still require engineers to possess strong experience in hardware architecture design. For task-specific hardware accelerator design in edge-side RSP, the key challenges identified in this paper are as follows: (1) There is a lack of methods for describing and optimizing the computational characteristics of task dataflow; (2) There is a lack of a bridge between software and hardware, making it difficult to realize software hardware co-design; (3) There is a lack of optimal search strategies for mapping from signal processing tasks to DSA architectures. To solve the above problems, this paper proposes SpChipADF, a DSA design framework for signal processing. The fundamental novelty of this work is that, unlike existing DSA, HLS, and dataflow-based frameworks, SpChipADF introduces a task-driven automatic accelerator design methodology that enables hardware architecture exploration at early design stages without requiring extensive hardware design expertise. This framework first establishes a cross-layer synchronous dataflow graph model based on the characteristics of streaming computing in signal processing. Then, it builds the top-level DSA heterogeneous system architecture and reconfigurable accelerator architecture, and designs reconfigurable computing modules and structural components to support flexible hardware dataflow reconfiguration. Finally, it maps the cross-layer synchronous dataflow graph to the reconfigurable dataflow architecture through heuristic search to achieve performance-resource co-optimization.

The research content of this paper is arranged as follows. Section 2 introduces the basic knowledge of signal processing architecture design and the existing problems; Section 3 introduces the proposed domain-specific accelerator design framework; Section 4 takes the *w*K SAR imaging algorithm as an example to conduct experimental verification and analysis on the proposed framework; Section 5 discusses and summarizes the research content and related results of the paper.

## 2. Preliminaries and Problem Analysis

This section first introduces the basic knowledge of DSA architecture design for RSP, including typical signal processing tasks and their computational characteristics. Then, based on these computational characteristics, a top-level DSA architecture for signal processing is proposed. Finally, through analysis, the key problems of domain-specific accelerator design that need to be solved urgently are presented.

### 2.1. Algorithmic Flow and Computational Characteristics of Typical RSP

**Figure 3:**
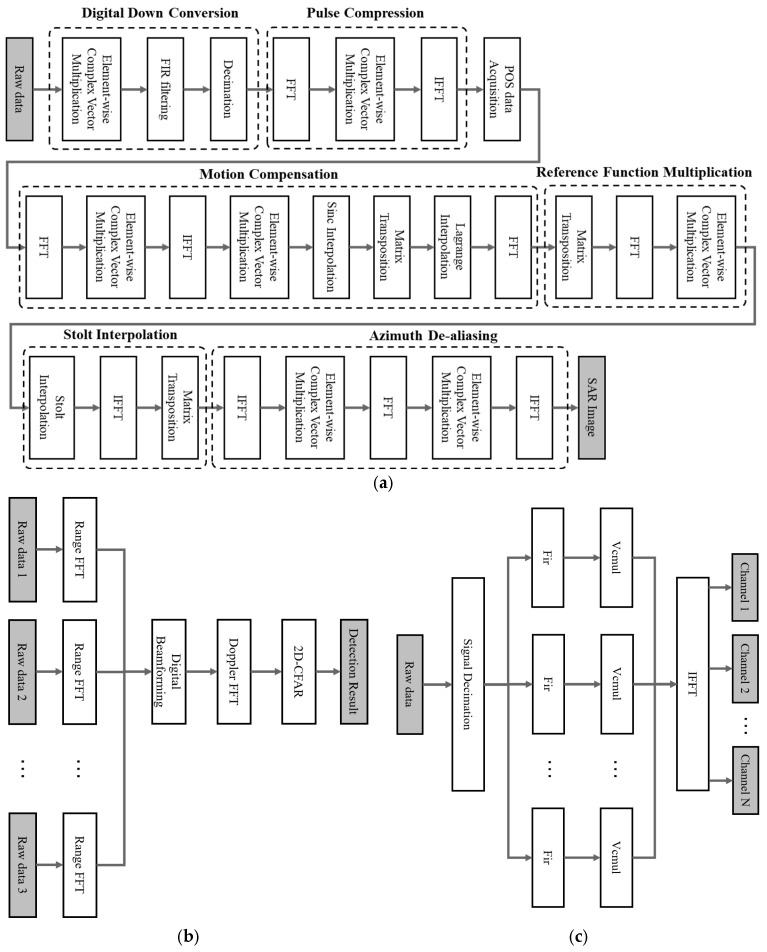
Flowchart of a typical signal processing algorithm. (**a**) SAR ωK Imaging Algorithm. (**b**) MIMO-DBF Imaging Algorithm. (**c**) Digital Channelization Algorithm.

In application scenarios such as target detection, intelligent transportation, and signal reconnaissance, typical radar signal processing algorithms include SAR imaging, Multiple-Input Multiple-Output Digital Beamforming (MIMO-DBF) imaging, and digital channelization. For lightweight UAV SAR imaging, the algorithmic flowchart is shown in Figure 3a. The main steps consist of digital down-conversion, motion compensation, reference function multiplication, Stolt interpolation, and azimuth de-aliasing. Through these procedures, the raw radar echo data are transformed into focused images that represent the electromagnetic scattering characteristics of the target scene. For MIMO-DBF imaging, the corresponding flowchart is illustrated in Figure 3b. Key operations include range-dimension FFT, digital beamforming, Doppler-dimension FFT, and 2D-CFAR detection. This process converts multi-channel radar echoes into images containing target position and velocity information, supporting high-resolution spatial sensing. The digital channelization algorithm, depicted in Figure 3c, performs decimation, filtering, element-wise vector complex multiplication (Vcmul), and parallel IFFT. It divides wideband radar echo data into several subbands, enabling fast detection and analysis of signals in frequency bands of interest.

Analyzing the algorithms above, we can see that signal processing algorithms mainly process electromagnetic wave data to obtain image information or target information of the scene. Typical real-time RSP algorithms are mainly composed of processing modules such as FFT, FIR filtering, interpolation, matrix multiplication, and matrix transposition. Moreover, their signal data streams present a data-independent and linear processing chain. The computational characteristics of signal processing algorithms can be summarized as follows [9,27]:

- It is tightly coupled with analog-to-digital (ADC) converters and digital-to-analog (DAC) converters;
- It is composed of a series of basic DSP operations;
- It is organized as a data-independent linear processing chain;
- It has deterministic and regular data streams;
- It has few branches and no interrupts;
- It requires a large dynamic data range;
- It exhibits alternating horizontal and vertical memory access patterns.

Furthermore, the algorithm flowchart indicates that SAR imaging constitutes a relatively complex RSP task, whose architectural design entails greater challenges compared with other algorithms. Hence, SAR imaging is primarily employed as the representative case in the subsequent research and validation presented in this paper.

### 2.2. Top-Level Digital System Architecture

**Figure 4:**
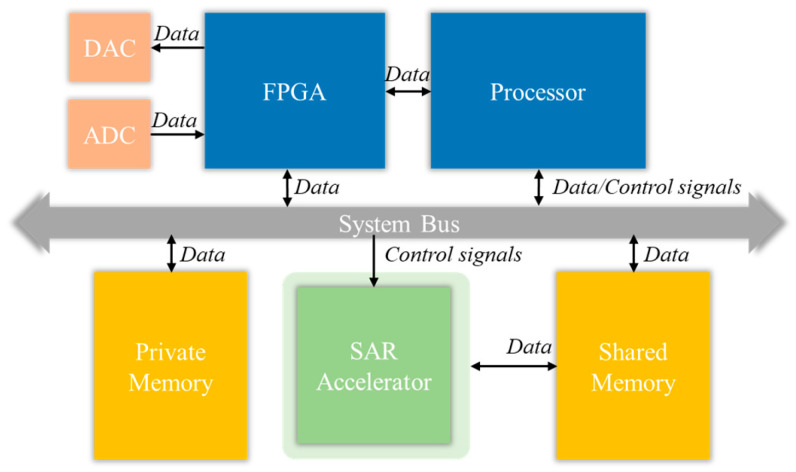
Schematic diagram of the top-level system architecture of signal processing DSA.

In response to the unique characteristics of RSP algorithms, namely high throughput, low latency, and deterministic data flow, traditional general-purpose processor architectures often struggle to meet their stringent requirements for real-time performance and energy efficiency. Therefore, this system adopts a domain-specific heterogeneous architecture design, as shown in Figure 4. The core of this architecture lies in the specialization and parallelization of hardware resources to accurately match the computational mode of RSP algorithms. RSP chain links comprise numerous FFT operations and matrix computations, exhibiting high computational density and predictable data flow patterns. Thus, dedicated hardware accelerators are introduced into the architecture, such as the SAR imaging hardware accelerator. It can efficiently execute specific DSP primitives in a pipeline manner, significantly reducing power consumption while ensuring processing speed.

From the perspective of system composition, at the front-end of the data path, the ADC and DAC are responsible for the conversion between analog and digital signals, while the FPGA acts as the core of high-speed data transfer and pre-processing, undertaking the tasks of real-time data stream caching and interface adaptation, which ensures the continuity of data acquisition. At the control level, the CPU is responsible for running the operating system, scheduling tasks, and configuring accelerator parameters. It issues control commands through the system bus, achieving flexible management and control of the entire system. At the computing core layer, hardware is solidified for specific SAR imaging algorithms, which greatly improves the computing power density. In addition, a hierarchical storage system consisting of private memory and shared memory is designed in the architecture. The former is used for high-speed caching inside the accelerator to reduce access latency, while the latter is used for data exchange and result storage among different processing units, thereby optimizing the overall data transfer efficiency.

### 2.3. Problem

**Figure 5:**
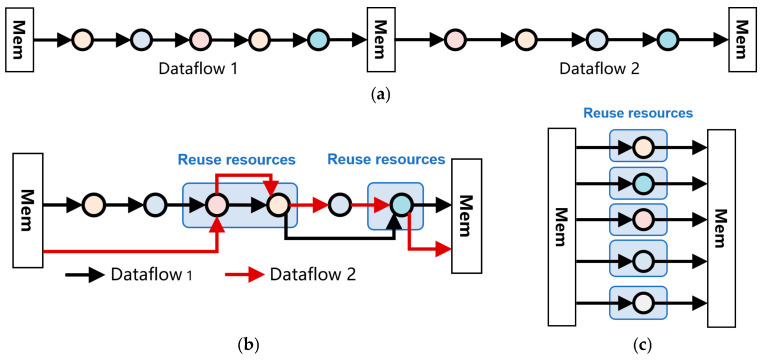
Signal processing task data flow graph decomposition and merging example. (**a**) Original Data Flow Graph. (**b**) Partial Resource Reuse Data Flow Graph. (**c**) Complete Resource Reuse Data Flow Diagram.

To fully exploit the synergistic advantages of DSA in resource efficiency and execution performance, systematic optimization strategies must be implemented at the accelerator architecture level. The core objective is to maximize the utilization of logic gate resources while strictly meeting task latency constraints. This optimization process is achieved through hierarchical decomposition of the data-flow graph and resource-reuse mechanisms. Figure 5a shows the original task data-flow graph, where nodes and edges form the initial computation and data-dependency relationships. Figure 5b introduces partial resource reuse. By identifying shareable operators or storage units, it reduces redundant hardware instantiation while preserving critical paths. Figure 5c evolves into a complete resource-reuse data-flow graph. Through structured reorganization and parallel scheduling, multiple task flows share the same set of computing and storage resources, thereby forming a highly compact accelerator topology in logic. This evolution from the original graph to the resource-reuse graph essentially balances “computation density” and “resource occupation”. Without sacrificing throughput or increasing latency, it significantly reduces the overall logic gate scale through cross-task data-flow alignment and hardware module reuse.

To achieve the optimal trade-off between resources and computational speed, this paper establishes a multi-objective optimization model for the exploration of hardware accelerator architecture design space,

(1) $$\underset{x}{\min}R\left(x\right)=\gamma_{1}\cdot A\left(x\right)+\gamma_{2}\cdot L(x),\;Subject\;to\;L\left(x\right)\leq L_{max}\;\;\;\;\;x\in T,\;\;\;\;\;\;\;\;\;\;$$

where $\lambda_{1},\lambda_{2}$ are the weighting coefficients for resources and latency, respectively, x is the decision variable, representing the architecture parameters from the original data-flow graph to the final resource-reused data-flow graph, T is the feasible domain of the architecture, $L\left(x\right)$ is the computational latency function, $L_{max}$ is the upper limit of computational latency, and $A\left(x\right)$ is the estimated function of the architecture’s logic gate resources. This model is a multi-objective optimization problem with latency upper-bound constraints. It aims to minimize both the resource cost $\;A\left(x\right)$ and the computational latency  L(x) within the feasible domain T under the premise that $\;L\left(x\right)\leq L_{max}$. Since these two objectives often conflict with each other, the optimal solutions form the Pareto front, which reflects the optimal design boundary of the DSA architecture in the “performance-resource” trade-off space.

Based on the above-established multi-objective optimization model for the exploration of the hardware accelerator architecture design space, this paper further identifies three key challenges in the process of implementing this model. These challenges constitute the core difficulties of automatic DSA design. Firstly, there is the semantic gap problem between task description and hardware mapping. Existing algorithm descriptions often lack explicit characterization of underlying hardware resources, making it difficult to directly guide the generation and mapping of hardware architectures. Secondly, there is the dimension-disaster problem of architecture search space. Due to the diversity of signal processing algorithms, the potential combinations of hardware architectures grow exponentially. Without targeted guidance, exhaustive search becomes infeasible. It is necessary to define the architecture design space and use directional search methods to improve design efficiency. Finally, after obtaining the final top-level accelerator architecture, it is necessary to establish the hardware description of the top-level system and conduct FPGA prototype verification to ensure consistency between the algorithm and the functionality of the hardware architecture.

## 3. Design Framework for Reconfigurable Hardware Architecture in RSP

To address the aforementioned challenges, this paper proposes SpChipADF, a domain-specific accelerator automatic design framework for signal processing. This section details each part of SpChipADF.

### 3.1. Overview of the Framework

**Figure 6:**
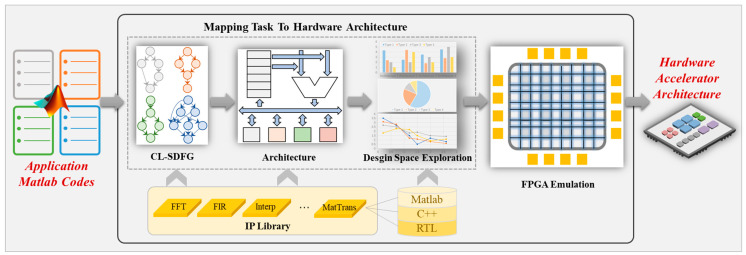
SpChipADF framework.

The core process of the proposed SpChipADF for signal processing is shown in Figure 6, and it mainly consists of four stages. Firstly, the input high-level signal processing algorithm (typically in the form of MATLAB 2021 code) is parsed and converted into a Cross-Layer Synchronous Data-Flow Graph (CL-SDFG). The CL-SDFG explicitly characterizes the data dependencies and parallelism among computational nodes, bridging the semantic gap between task description and hardware mapping. It provides a unified and fine-grained computational model for the subsequent architecture-level design space exploration. Secondly, design space exploration is carried out on the pre-defined reconfigurable data-flow architecture template. This template is composed of configurable processing units, storage modules, and interconnection structures. Its legal combination rules originate from the inherent data-flow characteristics of signal processing algorithms, which can constrain the originally huge random search space into a compact and feasible design domain. In this stage, the framework automatically adjusts the architecture parameters to generate multiple sets of candidate architectures. Thirdly, based on constraints such as the maximum processing time tolerable by the task and the maximum logic gate resources available for the hardware accelerator, the allocation of each operator in the data flow is optimized. Performance evaluation indicators, such as the total latency of task mapping to the architecture and the number of logic gate resources used by the architecture, are obtained to screen out better architecture schemes. Fourthly, according to the optimized architecture scheme, the top-level RTL connection relationship description of the hardware is generated. The framework maps the computational nodes in the CL-SDFG to the high-performance hardware implementations in the IP core library and automatically completes the data and control signal interconnection among various modules. It can quickly generate synthesizable FPGA top-level RTL code, laying a foundation for the subsequent functional and performance verification. Finally, simulation verification is performed on the FPGA platform. By actually running the generated hardware design, the functional consistency between the algorithm and the hardware acceleration architecture is verified. Moreover, key indicators such as throughput, resource utilization, and end-to-end latency are further extracted to confirm that the generated architecture meets the design objectives. The implementation details of each part of the framework are introduced in the following sections.

### 3.2. Cross-Level Synchronous Data-Flow Graph (CL-SDFG) Task Description Model

**Figure 7:**
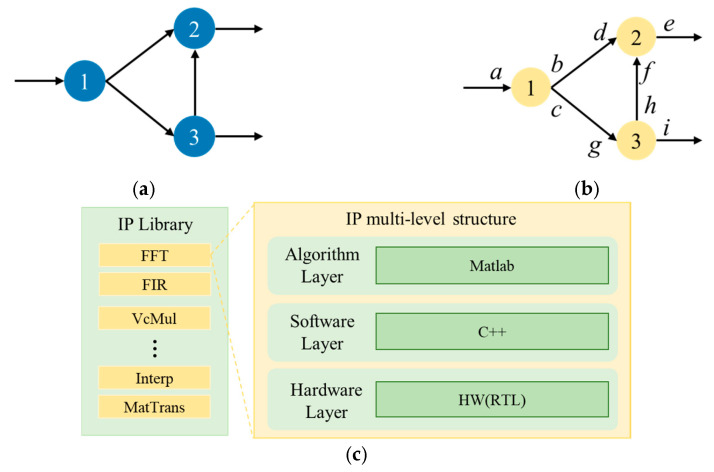
Directed-acyclic DFG. (**a**) Traditional DFG. (**b**) SDFG. (**c**) Operator library and hierarchical multi-level operator stack.

When tailoring a chiplet-based system architecture for RSP, the primary task is to construct a strict domain-specific model of the target algorithm. This task model then serves as a formal specification, driving all subsequent design decisions to evolve the architecture in a way that meets both application functionality and non-functional requirements [12]. The algorithm program can be jointly represented by a DFG and a Control-Flow Graph (CFG): the DFG explicitly declares the data-dependency relationships between operation nodes, while the CFG captures the control-flow execution order and transitions triggered by conditional branches, loops, and call sites [28]. The DFG is denoted as G(V,E), a directed acyclic graph that captures the data flow within basic blocks. The vertex set V(G) corresponds to basic operations, and the edge set E(G) encodes their direct data dependencies. To interact with surrounding modules, *G* is extended by adding a set of input/output ports $V_{+}$ and connecting these ports to the relevant edges $E_{+}\;$of the internal nodes V(G), resulting in $G_{+}\left(V\cup V_{+},E\cup E_{+}\right)$, as shown in Figure 7a.

Owing to the characteristics described in the previous subsection (RSP algorithms consist of basic DSP operations, have few control branches, and are essentially data-flow dominated), they can be modeled as a block diagram, i.e., a Large-Grain DFG (LG-DFG). In the LG-DFG, directed arcs connect atomic or coarse-grained nodes (such as adders, FFTs, etc.), and these nodes exchange infinitely long sample streams; the granularity of the nodes determines the exploitable parallelism, and the nodes can be hierarchically expanded into sub-graphs. Thus, a single graph can exhibit the data path, concurrency, and nested structure of the signal processing algorithm, which naturally aligns with DSP design practices [29].

To better align with the pipelined processing characteristics of signal processing, the Synchronous DFG (SDFG) refines the LG-DFG by associating a fixed token production/consumption rate with each node [17,29], as shown in Figure 7b. In the SDFG model, a module is triggered for execution only when each input edge contains a known fixed number of tokens; after being triggered, it precisely consumes the corresponding number of new samples from each input path. When analyzing this graph, if the number of samples consumed at each input port and the number of samples produced at each output port can be determined, the module is synchronous. As shown in Figure 7b, these fixed-rate annotations are placed next to the corresponding ports and become part of the module interface definition. The SDFG model ensures bit-level portability: as long as the semantics of the sample data stream remain unchanged, the same graph can be simulated on a single core, run on multiple cores, synthesized into dedicated hardware, or compiled into VLSI chips without altering the results [23,30]. To use the SDFG as a bridge from algorithm tasks to hardware implementation, we replace each node in the SDFG with a multi-level operator (MLO) customized from the signal processing algorithm and refined from the IP library. The SDFG with MLOs is called the CL-SDFG. As shown in Figure 7c, each MLO is described by three abstract layers, which have the same data interface to the outside while hiding internal implementation details:

- Algorithm layer (MATLAB): floating-point reference model;
- Software layer (C++): bit-accurate fixed-point model;
- Hardware layer (RTL): cycle-accurate, synthesizable Verilog model.

In the IP library, coarse-grained operators enable the CL-SDFG to be redirected to multi-core DSPs, FPGAs, or hardened VLSI macro-units without changing the semantics of the data stream. Therefore, the CL-SDFG has the following characteristics: (1) it is bit-accurate in structure; (2) it can describe data-flow characteristics and support static scheduling; (3) it can be gradually refined into the final chip-specific implementation.

To export a CL-SDFG from radar-signal-processing MATLAB code, we impose a light-weight syntax and implement a four-stage front-end. The restriction guarantees that every control branch is mapped to a conditional actor and every loop is unrolled or expressed as a vectorized function; only function calls and assignments may appear at the top level. The rules are:

(1) Function header:
function [result_1,result_2,…] = **moudleName**(…,…,…,…)end

(2) Function call:
[r1, r1, …] = **functionName**(a1, a1, …)

(3) Two-way conditional (higher arities are cascaded):
function y = **mux2to1**(x1, x2, sel)if sel  y = x1;else  y = x2;endend

(4) Loops are forbidden at the top level; they must be hidden inside functions or replaced by array operators.

A lexical analyzer written in Python 3.8.4 performs the following steps:

Step 1: Regular expressions tokenize the source file and label every construct that matches rules 1–4.

Step 2: Each statement is decomposed into triples (inputs, function, outputs). Typical patterns are as follows.
[out1, out2] = **funcA**(in1, in2, in3); // multi-returnout1 = **funcA**(in1, in2, in3);     // single-returnout1 = in1;               // direct copy


Step 3: One node is created for every function instance; edges are drawn from producer outputs to consumer inputs. Conditionals become mux/demux actors with boolean control ports. The result is a directed acyclic graph whose nodes obey the SDF semantics (fixed production/consumption rates).

### 3.3. Reconfigurable Architecture Templates and Component Design

**Figure 8:**
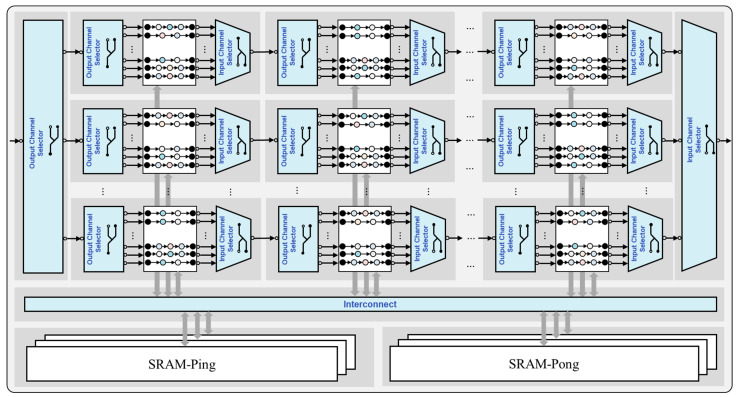
Template for a domain-specific reconfigurable dataflow hardware accelerator for signal processing.

The domain-specific reconfigurable data-flow (RDF) hardware accelerator architecture template for signal processing, as shown in Figure 8, achieves deep adaptation and innovative breakthroughs for streaming signal processing scenarios through the dynamic collaboration between switching components and reusable signal processing IPs. Its design core lies in utilizing the switching network composed of Output Channel Selectors (OCS) and Input Channel Selectors (ICS) to flexibly reconfigure different data flows: if two data flows can be mapped to continuous flows on the same path through the switch topology, they share the same processing row (i.e., the same set of IP instances); if reconfiguration is not possible, the instantiation of a new row is triggered, thereby dynamically expanding the processing capability in the spatial dimension. OCS can route the input data stream to different output ports, while ICS can select and forward data streams from different input ports.

As can be seen from the figure, the entire architecture is distributed in a grid-like manner. Horizontally, there are multiple rows of processing units, and vertically, there are multiple columns of channel selectors, forming a regular reconfigurable array structure. Each row contains multiple series-connected units of “OCS → Signal Processing IP Block → ICS”. Inside the signal processing IP block, there are several parallel channels (represented by circles and connecting lines to indicate data-flow paths), which support multi-channel concurrent processing. The channel selectors adopt switch symbols, indicating that they have data routing and reconfiguration capabilities. Data flows enter from the left-side input terminal, are distributed to the corresponding IP rows by the OCS, and then are aggregated and output to the right-side by the ICS. In the middle, the bottom Interconnect bus interacts with the SRAM-Ping/SRAM-Pong double-buffered storage module to realize the pipelining of data prefetching and processing.

This architecture realizes the dynamic aggregation of reusable IPs through the routing of switching components, constructing a “data-driven reconfigurable pipeline” for streaming signal processing. While maintaining efficient hardware reuse and low power consumption, it achieves adaptive support for complex and changeable dataflows.

**Figure 9:**
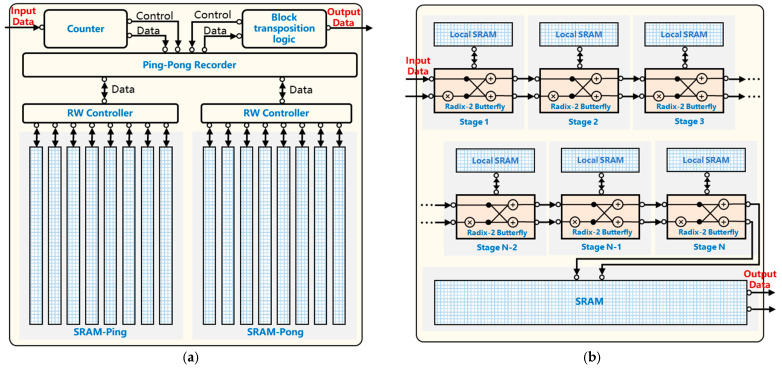

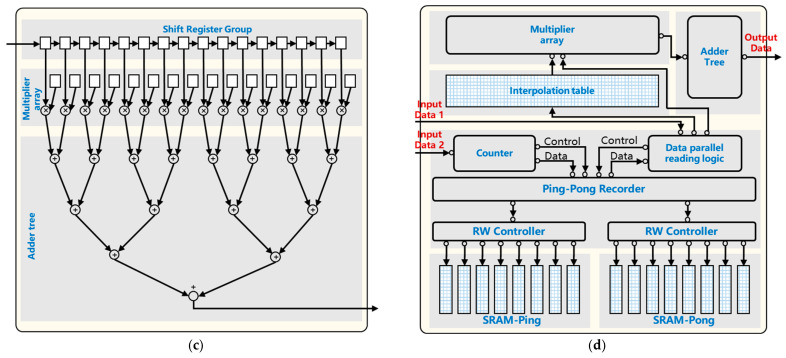
Signal processor specific domain reusable typical operator IP component. (**a**) Matrix transpose (MatTrans). (**b**) FFT. (**c**) FIR. (**d**) Interpolation (Interp).

By integrating typical pipeline IP components such as Matrix Transposition (MatTrans), Fast Fourier Transform (FFT), Finite Impulse Response filtering (FIR), and Interpolation (Interp), this paper enables modular, reusable, and dynamically reconfigurable support for signal processing algorithms, as illustrated in Figure 9. All these IPs are designed with a fully pipelined architecture, featuring high throughput and low latency. Their core advantage lies in the flexible scheduling and combination across different data flows via switch components, which dynamically adapt to the row-column structure of the “reconfigurable data-flow hardware accelerator template” in Figure 7. This achieves data-flow-driven on-demand occupation and aggregation of hardware resources. The detailed introduction is as follows.

1. MatTrans

As shown in Figure 9a, the data operation completed by the MatTrans IP can be expressed as: given an input matrix $A\in R^{M\times N}$, the output is its transpose matrix $A^{T}\in R^{M\times N}$, i.e., $A_{i,j}^{T}=A_{j,i}$. The architecture includes a counter, control logic, block transposition logic, Ping-Pong recorder, and dual-port SRAM (SRAM-Ping/SRAM-Pong) with read-write controllers. Data is alternately read and written through the Ping-Pong buffering mechanism, and the block transposition logic completes sub-matrix rearrangement locally, avoiding global data movement. The advantage of this design is to realize pipelined transposition through double buffering, reduce idle cycles, and simultaneously alleviate the storage bandwidth pressure through block-level operations. This adapts to the frequent matrix rearrangement requirements in SAR imaging.

2. FFT

The FFT IP (Figure 9b) implements the efficient computation of the Discrete Fourier Transform (DFT), and its operation can be abstracted as:

(2) $$X\left[k\right]=\sum_{n=0}^{N-1}x[n]\cdot e^{-\frac{j2\pi kn}{N}},$$

where x[n] is the time-domain input, and  X[k] is the frequency-domain output. The architecture adopts a multi-stage cascade of Radix-2 Butterfly computing units. Each stage contains local SRAM to store intermediate data, and the final result is output through global SRAM. The Butterfly units are interconnected via data paths to support pipelined parallel execution. The innovation of this design lies in decomposing the FFT into reusable Butterfly modules. Through local staged pipelined scheduling, high throughput and low power consumption are achieved, which is particularly suitable for the real-time requirements of large-scale frequency-domain processing in SAR imaging.

3. FIR

The FIR filter IP (Figure 9c) performs linear time-invariant filtering. Its output is given by:

(3) $$y[n]=\sum_{k=0}^{K}h[k]\cdot x[n-k],$$

where h[k] is the filter coefficient and x[n] is the input sequence. The architecture consists of a Shift Register Group, a Multiplier Array, and an Adder Tree. Data is passed level by level through the shift register, multiplied in parallel with the coefficients, and then accumulated and output by the adder tree. This design uses a fully pipelined structure and parallel multiply-accumulate units to achieve high-throughput filtering. At the same time, it reduces hardware overhead through register reuse. It is suitable for matched filtering and clutter suppression scenarios in SAR imaging.

4. Interp

The Interpolation IP (Figure 9d) realizes data sampling rate increase or coordinate remapping. Its operation can be described as:

(4) $$y\left[m\right]=\sum_{k}x\left[k\right]\cdot f(k,m),$$

where f(k,m) is the interpolation basis function (such as sinc or polynomial). The architecture includes an Interpolation Table, a Multiplier Array, an Adder Tree, a counter, control logic, a data parallel read logic, a Ping-Pong recorder, and a dual-port SRAM with a read-write controller. The interpolation table stores the pre-calculated basis function values. The multiplier array and adder tree complete the weighted summation. Data is read and written through the Ping-Pong buffer to realize pipeline operation. This design supports high-precision interpolation and high-throughput output. It is suitable for the requirements of point target refinement and image resampling in SAR imaging.

The above operator IPs are implemented and verified on FPGA in this paper. The resource statistics of each IP are shown in Table 1. In FPGA/ASIC design, according to relevant Xilinx manuals [31], 1 LUT6 ≈ 1.25 GE, 1 flip-flop (D-FF) ≈ 6 GE, 1 36 Kb BRAM (Xilinx UltraScale+) ≈ 100,000 GE, and 1 DSP Slice (27 × 18 multiplier + accumulator) ≈ 50,000 GE. The simplified model for estimating the number of logic gates used in ASIC from FPGA resources is as follows:

(5) $$N_{GE}=N_{LUT}\times 1.25+N_{FF}\times 6+N_{BRAM}\times 100,000+N_{DSP}\times 50,000.$$

### 3.4. Architecture Design Space Exploration

This section focuses on how to efficiently map the CL-SDFG to the proposed reconfigurable data-flow hardware accelerator template (Figure 8). The core of the mapping process lies in segmenting and merging the CL-SDFG based on the switch-reconfigurable characteristics of the architectural template, and then assigning the segments to a limited number of reconfigurable processing rows. This forms a combinatorial optimization problem with multiple constraints. The optimization objective is to minimize the amount of resources in the final hardware implementation under the premise of meeting hardware resource and task execution time constraints, so as to achieve a design with high resource utilization. This section will conduct a more detailed modeling of Equation (1) in Section 2.

The CL-SDFG to be mapped can be represented as G(V,E), where $V=\{v_{1},v_{2},...,v_{n}\}$ is the set of computational nodes, and each directed edge $e_{ij}\in E$ represents the data dependency and flow from node $v_{i}$ to $v_{j}$. The target architecture provides M reconfigurable processing rows, and the set of rows is denoted as $R=\{R_{1},R_{2},...,R_{M}\}$. The mapping needs to follow the following rules: (1) *G* can be segmented to form a set of subgraphs $\left\{G_{s}\left(V_{s},E_{s}\right)\right\}$, and adjacent subgraphs are merged according to whether the data flow can be reconfigured into the same path through the switch; (2) If a subgraph contains a Matrix Transposition (MatTrans) node, the data flow must be interrupted, and the data needs to be written back to the shared memory for rearrangement. Then the subsequent subgraph needs to start processing from a new row; (3) The delay required for task mapping to the hardware structure needs to be less than the set threshold $T_{th}$.

Based on this, we establish the following optimization model. Define the decision variable $\;x_{i,m}\in \{0,1\}$, which indicates whether node $v_{i}$ is mapped to $R_{m}$. Define the set of segmentation points, which determines the division of subgraphs. The optimization objective is to minimize the resource redundancy, which can be formalized as:

(6) $$\underset{x}{\min}R\left(x\right)=\gamma_{1}\cdot A\left(x\right)+\gamma_{2}\cdot L(G,x),$$

where $A(x)=\sum_{v_{i}\in V}x_{i,m}\cdot r(v_{i})$ is the total amount of resources occupied by $R_{m}$, $r(v_{i})$ is the resource requirement of node${\;v}_{i}$, *L*(***x***) represents the delay of mapping the CL-SDFG *G*(*V*,*E*) to the hardware architecture ***x***, and $\gamma_{1}$ and $\gamma_{2}$ are user-defined weighting parameters that reflect the relative emphasis on latency and resource requirements.

The model needs to satisfy the following constraints:

- Matrix Transposition Interruption Constraint: each $R_{m}$ can contain at most one MatTrans operator;
- End-to-End Delay Constraint: the total task execution time must be less than the given threshold $T_{th}$, i.e., $L\left(G,x\right)<T_{th}$.

For $L\left(G,x\right)$, first, the hardware architecture topology graph $G_{x}(V_{x},E_{x})$ can be constructed from the decision variable ***x***. According to the data continuity of the architecture and the task, G(V,E) is divided into *K* subgraphs ${\left\{G_{s}^{k}\left(V_{s}^{k},E_{s}^{k}\right)\right\}}_{k=1}^{K}$. Find the longest path $P_{x}^{k}$ of $G_{s}^{k}$ in $G_{x}(V_{x},E_{x})$:

(7) $$P_{x}^{k}\left(G,x\right)=arg\underset{P\in P\left(G_{x},G_{s}^{k}\right)}{\min}\left(l\left(P\right)\right)\;$$

where $P\left(G_{x},G_{s}^{k}\right)$ is the set of all paths that cover the data flow of $G_{s}^{k}$, and l(P) represents the length of path *P*. Then, the delay of each data flow is divided into two parts: data reading and processing. Finally, the processing delays of all data flows are summed up to obtain the final delay evaluation result, which can be expressed as:

(8) $$L\left(G,x\right)=\sum_{i=1}^{K}\left(\frac{N_{k}}{\max\left(\eta B_{max},B\left(P_{x}^{k}\right)\right)}+\frac{N_{k}}{\eta B_{max}}\right)$$

where $N_{k}$ is the amount of data processed by the *k*-th data flow, η is the memory read—write efficiency, $B_{max}$ is the maximum theoretical read-write bandwidth of the memory, and $\;B(P_{x}^{k})$ is the bandwidth of path $P_{x}^{k}$. And η is related to the memory access mode of DDR. For DDR4, its memory access efficiency can be specifically expressed as [32]:

(9) $$\eta =\left\{\begin{array}{cc} 94, & Sequential\;Read\\ 89, & Sequential\;Write\\ 90, & Burst\;Read/Write\;Mix\\ 51, & Short\;Burst\;Read/Write\;Mix\\ 24, & Random\;Address\;Read/Write\;Mix \end{array}\right.$$

For $B\left(P_{x}^{k}\right)$, the bandwidth of a path is determined by the “bottleneck link”—the total bandwidth of the path equals the minimum bandwidth among all its adjacent links, which can be expressed as:

(10) $$B\left(P_{x}^{k}\right)=\underset{e\in P_{x}^{k}}{\min}B(e)$$

where B(e) denotes the bandwidth of edge $e=(v_{e}^{o},v_{e}^{i})$, which can be calculated based on the data generation rate and consumption rate of the two nodes connected by the edge. Specifically, $\;B\left(e\right)=\min\left(O\left(v_{e}^{o}\right),I\left(v_{e}^{i}\right)\right)\cdot f$, where $O\left(v_{e}^{o}\right)$ and $I\left(v_{e}^{i}\right)$ represent the data generation rate of node $v_{e}^{o}$ and the data consumption rate of node $v_{e}^{i}$, respectively, and f denotes the clock frequency.

**Figure 10:**
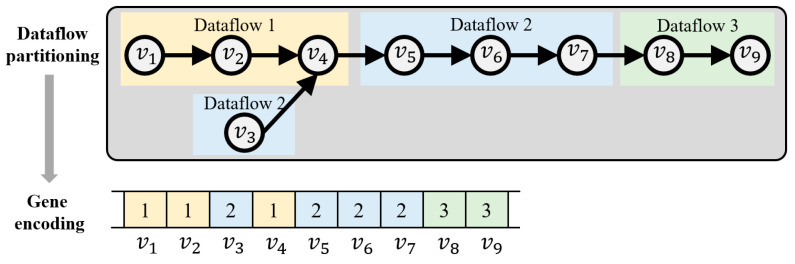
Gene encoding diagram.

The problem is a high-dimensional, discrete, and multi-constrained NP-hard joint optimization of resources and latency. Compared to other heuristic search methods, such as greedy algorithms and simulated annealing, the genetic algorithm offers stronger global search capability, better adaptability to discrete decisions, enhanced ability to balance multiple constraints, as well as greater robustness and scalability. It explores the solution space through population evolution, escapes local optima via crossover and mutation, and integrates constraints and objectives through a fitness function, thereby achieving a balance between time efficiency and solution quality. Accordingly, this paper employs an adapted genetic algorithm for solving the problem. To solve the problem using the genetic algorithm, the decision variable x is first encoded into genes. The principle of this encoding is illustrated in Figure 10, representing the process of mapping nodes to the dataflow.

Based on the above analysis, we propose a genetic algorithm for graph partitioning in reconfigurable dataflow architectures. The detailed procedure is outlined in Algorithm 1.

**Algorithm 1:** Graph-partitioning-driven Multi-objective Genetic Search For Reconfigurable Dataflow Architecture

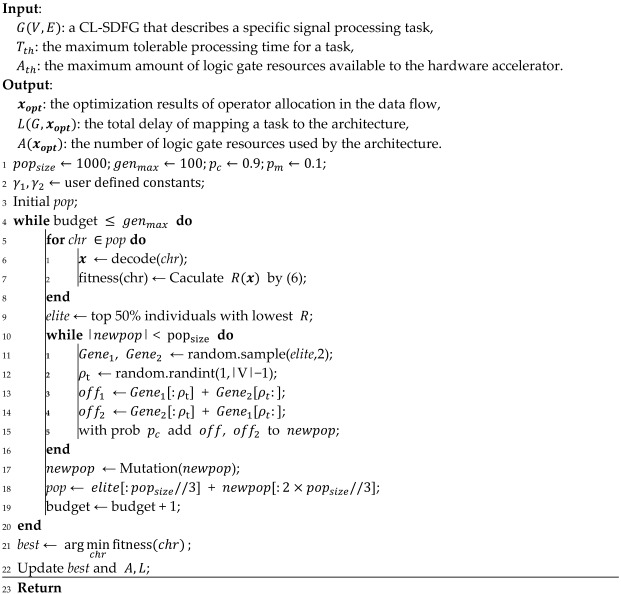



### 3.5. Hardware Architecture RTL Generation

After obtaining the optimal reconfigurable data-flow architecture, the next step is to generate the top-level hardware description to achieve the precise mapping from the algorithm-level data-flow graph to the physical hardware structure. As shown in Figure 10, the Hardware Top-Level Description Generator, as the core module, takes the optimized CL-SDFG structure and the reconfigurable architecture template as inputs. Combined with the pre-defined Reusable IP Library for DSP and the Reconfigurable Switch component, it automatically constructs the interconnection topology among various IP modules and completes the functional integrity verification. This process ensures that the finally generated hardware architecture not only meets the resource constraints and performance objectives, but also strictly preserves the data dependencies and functional semantics of the original CL-SDFG.

**Figure 11:**
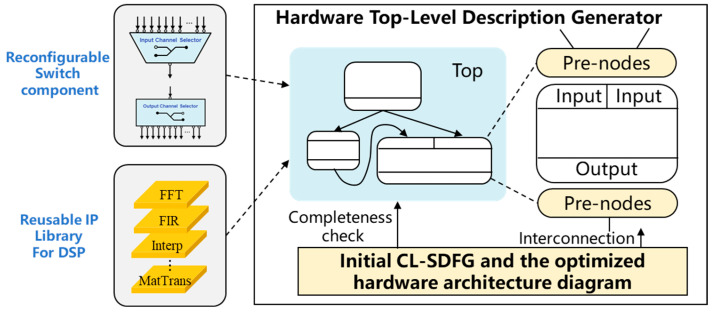
Schematic diagram of the top-level description of the hardware architecture.

Specifically, the top-level generator first parses the sub-SDFG carried by each processing row in the optimized architecture and the corresponding operator IP instances (such as FFT, FIR, Interp, MatTrans, etc.) and calls the parameterized descriptions of the corresponding modules from the IP library. At the same time, according to the reconfiguration requirements of the data-flow paths, it inserts reconfigurable switch components into each input/output port. Its structure is shown on the left side of Figure 11, including an ICS and an OCS, which are used to dynamically route data to the target IP or memory. Then, the generator instantiates each IP and switch in the Top-level module according to the connection relationship of the data-flow edges $e_{ij}\in E$ in the CL-SDFG, and connects them in topological order through the Interconnection network to form a complete hardware data-flow path.

After the interconnection is completed, the generator performs a Completeness check to verify whether all nodes and edges in the CL-SDFG have corresponding implementations in the hardware architecture, and whether the data-flow paths are free of breaks or conflicts. In particular, for operations such as matrix transposition, it is necessary to confirm whether its data rearrangement phase has been correctly mapped to the combined path of shared memory and switch control, so as to avoid functional errors caused by data-flow interruption. Finally, the generator outputs the top-level hardware description file that conforms to the HDL (such as Verilog/VHDL) specification, providing directly synthesizable design input for subsequent FPGA or ASIC implementation.

## 4. Experiments and Results

### 4.1. Experimental Setup

SAR imaging, a high-complexity radar signal processing (RSP) task, is taken as a test case in this section to verify the effectiveness of the proposed reconfigurable data-flow architecture and its design flow. Real SAR echo data are used for verification to ensure that the results can accurately reflect the performance of the proposed method in practical scenarios with complex clutter, noise, and massive data processing.

At the software level, a computer equipped with an AMD Ryzen 5 5800 H CPU is used to perform CL-SDFG modeling, architectural space exploration optimization algorithms, and pre-processing of real-measured SAR data. At the hardware implementation and verification level, the Xilinx ZCU102 development board is selected as the target platform. The ZCU102 is equipped with a Zynq UltraScale+ MPSoC, which has abundant programmable logic resources (including look-up tables, block RAM, DSP Slices, etc.). It can implement the hardware design generated by the top-level hardware description generator and conduct real-time SAR imaging processing verification.

### 4.2. Experimental Results

**Figure 12:**
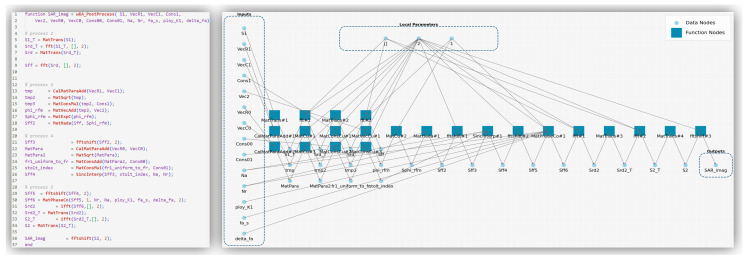
From MATLAB Implementation to CL-SDFG of SAR *w*KA.

This experiment takes the SAR imaging *w*K algorithm as an example and uses the proposed framework to conduct architecture design and optimization. First, based on its MATLAB code, a CL-SDFG model, as shown in Figure 12, is generated. It verifies that the CL-SDFG can accurately characterize the data dependencies and execution timing among various core operations in the algorithm and completely preserve the data-flow semantics from input data to the final imaging result. This provides a formalized and visual graph model for the subsequent design of reconfigurable hardware architectures.

**Figure 13:**
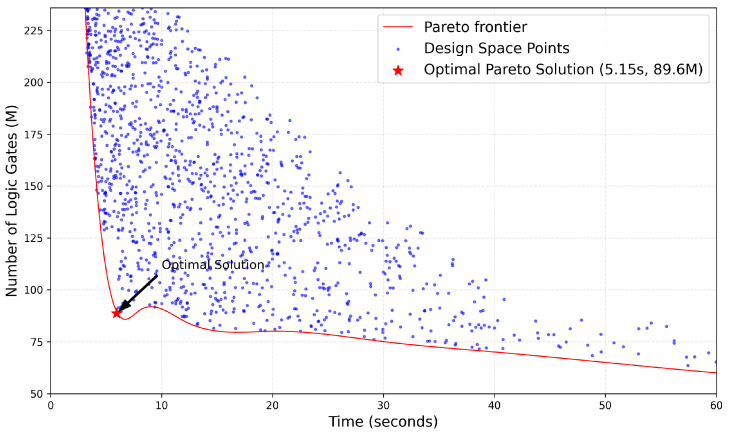
Architecture design space exploration results.

For the constructed CL-SDFG, this study conducts design space exploration work, aiming to balance two core design metrics: system time cost and logic gate scale. Through a heuristic search algorithm, the design space is traversed and optimized. Finally, the Pareto Frontier is obtained, which characterizes the set of all non-dominated solutions. In this set of solutions, both computational delay and the number of logic gates cannot be further optimized simultaneously. By applying equal weighting to latency and logic gate usage, the solution corresponding to the optimal value is selected as the Pareto optimal solution. In the visualization results of design space exploration, as shown in Figure 13, blue scatter points represent the sampling points of the design space, the red curve is the Pareto Frontier, and the red stars mark the Pareto optimal solution. The corresponding performance parameters are a time cost of 5.15 s and a logic gate scale of 89.6 million.

**Figure 14:**
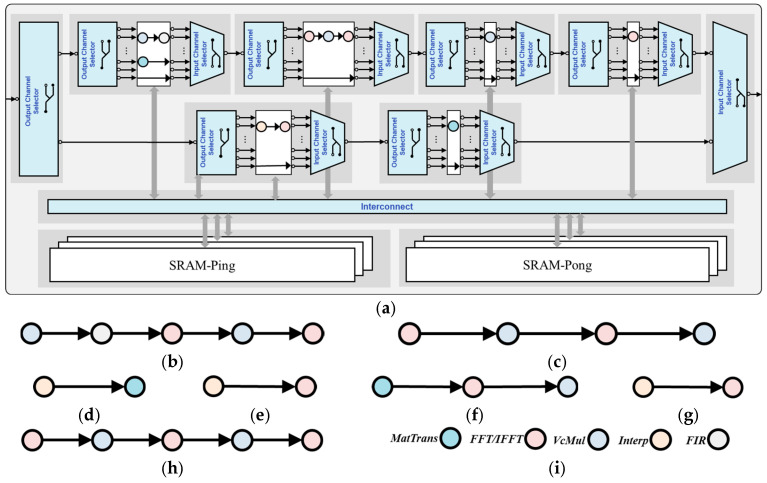
A reconfigurable dataflow accelerator architecture for SAR imaging generated by the proposed method. (**a**) Overall Architecture. (**b**) Dataflow 1. (**c**) Dataflow 2. (**d**) Dataflow 3. (**e**) Dataflow 4. (**f**) Dataflow 5. (**g**) Dataflow 6. (**h**) Dataflow 7. (**i**) Annotation corresponding to the operator IP.

The SAR imaging hardware accelerator architecture model corresponding to the Pareto-optimal point (5.15 s, 89.6 M) is shown in Figure 14. This architecture is designed for the algorithmic characteristics of SAR imaging and integrates functional nodes such as MatTrans, FFT/IFFT, vector multiplication (VcMul), Interp, and FIR. It supports multi-mode data processing through flexible configuration and interconnection mechanisms.

From the perspective of modular design, the ICS and OCS are responsible for routing and distributing data flow. The Interconnect module realizes high-speed data interaction among various functional nodes. The dual-port SRAM ensures pipelined data throughput through the ping-pong operation mechanism, avoiding storage bottlenecks. The core advantage of this architecture lies in its reconfigurable data-flow capability: by configuring different node connections and operation sequences, 7 customized data flows can be generated (as shown in Figure 14b–h). These data flows are executed sequentially according to the timing logic of SAR imaging processing and finally complete the entire processing process from raw echo data to SAR images.

Relying on the operator IP library, and with the automated flow of Xilinx Vitis tools, this paper realizes the automatic generation of RTL projects. This process verifies the accuracy of the proposed architecture design framework: from the functional module division and data-flow logic of the architectural model to the module instantiation, signal connection, and clock domain synchronization at the RTL level, all are highly consistent with the design intent. This ensures the accurate mapping of data paths and control logic among hardware modules.

Furthermore, this paper conducts physical implementation and functional verification of the proposed architecture on the FPGA platform. The resource statistics (Table 2) show that compared with the traditional method, the proposed architecture reduces the occupancy rates of key resources such as LUT, FF, BRAM, and DSP by 51.25%, 48.38%, 53.91%, and 48.10%, respectively, and the GE (equivalent gate count) is reduced by 51.4%, reflecting a significant resource optimization effect. The delay test (Table 3) compares the estimated delay and the measured delay of 7 reconfigurable data flows. The delay estimation error for the second dataflow is close to 30%. This error is believed to be caused by the deviation between the actual memory access efficiency and the ideal memory access efficiency. However, this deviation exhibits randomness, and after averaging, the overall estimation error is 0.03 s (approximately 0.5%). This result verifies the consistency between architectural design in performance prediction and engineering implementation, and ensures the real-time requirements of SAR imaging processing.

**Figure 15:**
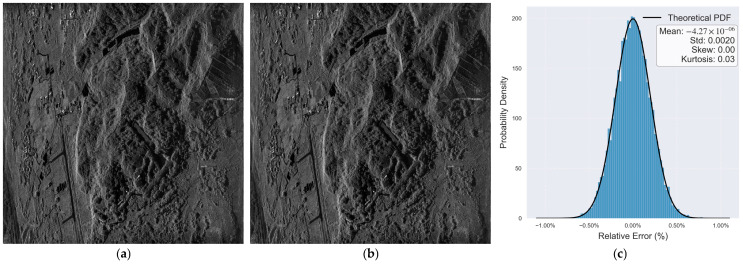
Validation results of a reconfigurable hardware accelerator architecture for SAR imaging. (**a**) MATLAB calculation results. (**b**) FPGA verification results. (**c**) Distribution of relative errors.

For SAR echo data with a Ku-band 32 K × 16 K scale, after processing, SAR images are generated. The data is obtained from a hexacopter UAV measuring 1.5 m × 1.5 m × 1 m in size, with a payload capacity of approximately 6 kg. By comparing with the processing results of MATLAB software (Figure 15a), the prototype verification results of the RTL of the hardware accelerator generated by the proposed architecture on the FPGA (Figure 15b), and analyzing the relative error distribution between the two (Figure 15c), the consistency verification of algorithm function and hardware implementation is completed.

The results show that the SAR image after hardware acceleration highly matches the MATLAB calculation result in visual texture and target contour. The relative error distribution closely fits the theoretical probability density curve. This verifies that the proposed reconfigurable hardware accelerator architecture can accurately reproduce the function and precision of the SAR imaging algorithm, and the hardware implementation does not introduce significant errors.

Meanwhile, this verification process also proves that the full-process design method, from algorithm modeling and architecture transformation to hardware prototype verification, can effectively guarantee the correctness and reliability of the SAR imaging algorithm on the hardware.

### 4.3. Result Analysis

Through heuristic design space exploration based on the CL-SDFG model, this study successfully locates the Pareto-optimal point (5.15 s, 89.6 M) under the dual constraints of time cost and logic gate scale. This solution is at a significant inflection point of the Pareto frontier, which not only meets the real-time requirement of SAR imaging processing but also controls the hardware resource consumption within a reasonable range, providing a performance-resource balance benchmark for subsequent architecture design. The “first steep then gentle” change trend of the Pareto frontier clearly reveals the law of diminishing marginal returns for time optimization and resource consumption.

The reconfigurable data-flow architecture (RDFA) built based on this optimal solution integrates core operator modules such as MatTrans and FFT/IFFT through modular integration and flexible interconnection, supporting efficient switching of 7 customized data flows. With the help of the Xilinx Vitis toolchain, RTL is automatically generated, verifying the accurate mapping from the architectural model to the hardware description. The clear distinction between Streaming Port (SP) and Streaming Connection (SC) interfaces, along with the well-designed data paths, ensures effective coordination between control logic and computational pipelines, providing a solid foundation for subsequent FPGA implementation.

In this paper, the authors manually designed a non-reconfigurable pipelined hardware accelerator architecture for SAR imaging, utilizing the operator IPs presented herein. The design primarily focused on pipeline optimization, without conducting further resource optimization. As shown in Table 4, compared with the traditional non-reconfigurable data-flow architecture, under the premise that the amount of data processed is the same (32 K × 16 K), the number of equivalent GE resources in the proposed reconfigurable architecture decreases from 217,911,896 to 105,854,136, with a reduction rate of 51.4%; the power consumption decreases from 28.5 W to 13.9 W, with a reduction rate of about 51.2%; the energy efficiency increases from 3.76 Mega Samples per Joule (MS/J) to 7.15 MS/J, with an increase of about 90.2%; the processing delay only increases by 0.05 s (from 5.10 s to 5.15 s), and the throughput slightly decreases from 100.4 MS/s to 99.4 MS/s, with negligible performance loss. Compared with the processing architecture based on reconfigurable computing [33], the RDFA designed by the proposed method demonstrates improvement across all metrics. Compared with the RDFA in Reference [11], the data processing capacity of the architecture in this paper increases from 8 K × 8K to 32 K × 16 K (a 400% increase), the number of equivalent GE resources increases from 33,649,000 to 105,854,136 (a 214.5% increase), but the energy efficiency still increases from 5.7 MS/J to 7.15 MS/J (a 25.4% increase), and the power consumption only increases from 3.8 W to 13.9 W (a 265.8% increase). Moreover, the advantages in processing delay (5.15 s vs. 2.97 s) and throughput (99.4 MS/s vs. 21.5 MS/s) are magnified with the increase in data volume, reflecting the superiority of energy efficiency and performance under large-scale data processing.

The collaborative design framework proposed in this study successfully closes the technical loop from algorithm optimization to hardware implementation. Experimental verification shows that this scheme achieves the best balance among performance, power consumption, and hardware cost in compute-intensive applications such as SAR imaging, providing a design paradigm that can be engineered for high-energy-efficient embedded signal processing systems.

## 5. Conclusions

To address the difficulty of balancing efficiency, resource utilization, flexibility, and development cycle in traditional hardware design under strict SWaP constraints for lightweight edge devices, this paper proposes an algorithm-hardware co-design framework based on the CL-SDFG. This framework is intended to address the core contradiction of “difficulty in achieving both low delay and low resource usage” in high-performance signal processing hardware design. The framework builds a unified formal model of algorithms and hardware and implements a full-chain toolset covering CL-SDFG modeling, multi-objective design space exploration, automatic generation of reconfigurable architectures, and RTL code conversion, which effectively bridges the gap between algorithm semantics and underlying hardware implementation. Validation results using SAR imaging as an example show that, for a data scale of 32 K × 16 K, the proposed method reduces the number of equivalent gates by 51.4% compared with traditional non-reconfigurable architectures. Compared with manually designed reconfigurable architectures, it increases the processing scale fourfold and improves energy efficiency by 25.4% under the automated design process, while significantly reducing development complexity. Although the current optimization effect is limited by the pre-defined IP library and the heuristic search capability, this paper provides a high-performance, low-power, and highly scalable hardware acceleration paradigm for compute-intensive edge signal processing applications and has important theoretical and engineering value for promoting the design of dedicated computing systems under strict SWaP constraints.

## Figures and Tables

**Table 1:** Reusable IP Hardware Implementation Resource Statistics.

	LUT(%)	FF(%)	BRAM(%)	DSP(%)	GE
FFT	2.52	2.22	5.76	2.38	15,420,868.8
Interp	9.72	7.50	8.77	5.60	29,997,048
FIR	5.92	3.82	2.14	2.58	11,199,052.8
MatTrans	0.98	0.63	7.02	0.00	10,273,255.2
Total	21.66	16.39	29.45	12.94	66,890,224.8

**Table 2:** SAR Imaging Hardware Accelerator Resource Statistics.

	LUT(%)	FF(%)	BRAM(%)	DSP(%)	GE
Traditional method	64.29	48.26	86.24	29.52	217,911,896
Proposed method	31.34	24.91	39.75	15.32	105,854,136
Reduced proportion(%)	51.25	48.38	53.91	48.10	51.4

**Table 3:** Validation experimental results of the evaluation model for processing delay.

	Estimated Computation Delay (s)	Measured Computational Delay (s)	Error (s)
Dataflow 1	1.360	1.320	+0.040
Dataflow 2	0.680	0.520	+0.160
Dataflow 3	0.350	0.370	−0.020
Dataflow 4	0.480	0.495	−0.015
Dataflow 5	0.660	0.645	0.016
Dataflow 6	0.510	0.550	−0.040
Dataflow 7	0.540	0.555	−0.015
Total	5.150	5.120	+0.030

**Table 4:** Metric Statistics Table of Reconfigurable Data Flow Architecture for SAR Imaging.

	Data Volume	Processing Latency (s)	Throughput (MS/s)	Equivalent GE Count	Power(W)	Energy Efficiency (MS/J)
Traditional Non-reconfigurable DFA	32 K × 16 K	5.10	**100.4**	217,911,896	28.5	3.76
RA in [33]	64 K × 64 K	85.90	47.7	62,971,165	30.6	1.56
RDFA in [11]	8 K × 8 K	**2.97**	21.5	33,649,000	3.8	5.70
The Proposed RDFA	32 K × 16 K	5.15	99.4	**105,854,136**	**13.9**	**7.15**

The bold text here indicates the optimal value in each column of the table.

## Data Availability

The data used to support the findings of this study are available from the corresponding author upon reasonable request. Due to privacy concerns, the data are not publicly available.
